# Adolescent HPV Vaccination: A U.S. and Global Perspective on Overcoming Barriers

**DOI:** 10.65638/2978-882x.2025.01.10

**Published:** 2025-12-24

**Authors:** Valeria Gutierrez Garcia, Rachel Mpanumpanu, Asha Ciara Sneed, Natalie Romero, Lunthita M. Duthely

**Affiliations:** 1 Massachusetts General Hospital and Harvard Medical School; 2 University of Miami School of Medicine, Obstetrics, Gynecology and Reproductive Sciences; 3 University of Maryland School of Medicine

**Keywords:** HPV, Human papillomavirus, Vaccination, Immunization disparities, Cancer prevention, Adolescent health, Global health equity

## Abstract

Human papillomavirus (HPV) is the most common sexually transmitted infection in the United States and a major contributor to preventable cancers, including cervical, anal, and oropharyngeal cancers, with consequences for individual and population wellbeing. Despite the availability of effective vaccines, HPV vaccination rates remain significantly lower than those for other adolescent immunizations. This health equity-focused editorial explores key factors contributing to low vaccine uptake, including age-based recommendations, testing limitations, affordability, and structural barriers to access. Regional and gender disparities within the U.S., coupled with stark global differences between high-income and low- and middle-income countries, highlight the urgent need for targeted strategies. Improving HPV vaccination coverage requires a multifaceted approach, combining public education, provider advocacy, and capacity building to ensure equitable access. Addressing these gaps is essential to reduce HPV-related disease burden, improve population wellbeing and advance global health equity.

## INTRODUCTION

Each August, the United States (U.S.) observes National Immunization Awareness Month (NIAM) to raise awareness about the importance of vaccinations in preventing serious illnesses [1]. Campaigns like NIAM remain critical for public health, especially as pathogens such as SARS-CoV-2 have reinforced the value of widespread immunization. During the SARS-CoV-2 Pandemic (Pandemic), vaccination rates surged; however, routine childhood immunizations have since fallen back to pre-pandemic levels, prompting renewed efforts through NIAM and online public health messaging to encourage parents and caregivers to vaccinate their children [2]. Among recommended childhood vaccines, HPV stands out for its persistently low uptake compared to others such as MMR and Tdap [3]. This pattern is concerning because HPV vaccination supports long-term wellbeing by preventing multiple cancers, including cervical, anal, and oropharyngeal cancers. While HPV vaccination rates in high-income countries like the U.S. exceed those in low- and middle-income countries (LMICs), disparities remain significant both domestically in the U.S. and globally [4]. These gaps suggest that awareness campaigns alone are insufficient, and more comprehensive strategies are needed to improve HPV vaccine coverage.

## UNDERSTANDING HPV RISK AND DISEASE BURDEN

HPV is the most common sexually transmitted infection (STI) in the U.S., yet many people see it as solely an STI [5]. Similar to other STIs, it often goes many years without creating any problematic effects. For the unlucky ones, though, HPV infection can lead to anogenital conditions like the development of genital warts or even some cancers [6]. Left untreated, HPV infection can cause cancers of the vagina, vulva, penis, cervix, anus, and oropharynx [7]. In 2019, it was estimated that 620,000 new cancer cases in women and 70,000 new cancer cases in men were caused by HPV, globally [8]. In the U.S., the incidence for cervical cancer was 7.6 cases per 100,000 women [9]. However, the greatest global burden of HPV-associated cancers is cervical cancer in LMICs. Overall, HPV-associated cancers account for 6.7% of all cancers in LMICs compared with 2.8% in high-income countries [10]. Cervical cancer is one of the few cancers caused by HPV that can be detected early, thanks to aggressive and regimented cervical cancer screenings, typically during a pap smear. Although this global burden is well documented, the impact of HPV for individual wellbeing remains unrecognized. Individuals with an HPV infection may experience mental health challenges, including distress related to the transmission and preventability of HPV [10]. Stigma surrounding sexual transmission is common, particularly in LMICs, where HPV is frequently associated with promiscuity, prostitution, and infidelity [11]. Individuals may also worry about increased cancer risk for sexual partners, or avoid sexual intimacy due to fear of transmission [10]. HPV is one of the few STIs with an available vaccine [12]. Why is it, then, that HPV vaccination rates in the U.S. are not higher?

## HEALTH EQUITY FRAMEWORK

The Health Equity Framework proposed by the Education Training Research Organization describes how health and education outcomes are shaped by complex interactions between individuals and their environments. The model defines health equity as having personal autonomy over one’s health and fair access to resources needed to achieve the best possible physical, emotional, and social wellbeing. The model describes health inequities as preventable differences in health outcomes that are related to social, economic and environmental conditions. The framework outlines four interconnected factors: relationships and networks, individual factors, physiological pathways, and systems of power. In this framework, health equity is promoted when these interacting factors are supported and maximized, while health inequities occur when structural and social barriers restrict them. Relationships and networks support health equity by fostering social connections that encourage healthy choices, while individual factors reflect how attitudes, skills and behaviors influence responses to social, economic and environmental conditions. Physiological pathways contribute to health equity by supporting physical, cognitive, and psychological functioning. Together with these factors, systems of power such as policies, processes and institutional practices determine access to resources and opportunities needed to support healthy lives [13]. The health equity framework provides a useful lens for understanding disparities in HPV vaccine uptake. Within the context of HPV vaccination efforts, this framework emphasizes that equitable vaccine coverage depends not only on individual choice, but also on supportive social networks, accessible and affordable services, and policies that reduce structural barriers.

## TESTING AND VACCINATION CHALLENGES

There are some caveats related to HPV testing and vaccination, which may contribute to low uptake in the U.S. Over 80% of people are infected with HPV at some point in their lives, and its frequent (though usually harmless) appearance in young people would cause worrisome, positive HPV tests [14]. It is recommended, therefore, to commence HPV testing after the age of 25 [15]. Accordingly, the best approach to reduce the risk of HPV infection is to take the preventative measure of early vaccination.

Until recently, the CDC only recommended HPV vaccination for individuals aged 11-26, based on evidence that the greatest benefit occurs before exposure and during peak immune responsiveness. While it would not be harmful, vaccination for people outside this age range was considered minimally beneficial, because HPV exposure has likely already happened [16]. This is still the case, but a recent report by the Advisory Committee on Immunization Practices (ACIP) extended the vaccination age to 45 years old, based on recent evidence that the benefit of HPV vaccines extends beyond the age of 26 [17-18]. Older adults who are not already vaccinated against HPV should assess their risk levels; they may be at risk for new HPV infection, and they could receive protection from types of HPV other than the one they may have been exposed to.

Public misconceptions can create barriers to wellbeing by making vaccination more challenging. Research has shown that providers are less likely to recommend HPV vaccine if they feel uncomfortable discussing sexual health, perceive parents as hesitant, or believe that their patients were at low risk. Similarly, patients are less likely to receive recommendations if they were younger, male, or from racial/ethnic minority [19]. Additionally, a cross-sectional survey of 153 medical providers across 23 African countries, found that while 83% of providers reported recommending HPV vaccine in their practice, only 37% indicated that the HPV vaccine was available at their facility [20]. In Malawi, a study conducted by Masamba Makanani *et al*. identified additional barriers to HPV vaccine uptake, including negative attitudes towards the vaccine, misconceptions arising from inadequate information and vaccine shortages [21].

## BARRIERS TO VACCINE ACCESS

Tenets of public health point to the necessity of resources needed to improve uptake of an intervention. The mere availability of a vaccine is not enough. The vaccine must be easily accessible as well. Common barriers to vaccination are affordability and knowledge, and access to transportation to travel to a vaccination site. For children, the impacts of these barriers have been mitigated, due to community-based programs that offer free or low-cost vaccines at convenient times and locations for families [22]. There are fewer programs for adults seeking vaccines however, whether for HPV or other illnesses, who must search for them on their own.

In the U.S., differences in insurance coverage can make it more challenging for adults to access HPV vaccine and may reduce vaccine uptake in this population. A study found that in most states, Medicaid plans cover HPV vaccination for adults aged 27-45 years, with 43 states providing coverage through age 45 without prior authorization; however, coverage mechanisms vary, some states require prior authorization, and Mississippi does not cover adults older than 26 years [23]. Therefore, uninsured adults or adults not in primary care, have to figure out if the vaccines they seek are offered at low or no cost. Online resources like the CDC’s guide on paying for vaccines are an available resource for adults, but they may be inadequate to improve vaccination uptake among adults in the U.S. [24]. These access barriers are closely related to the social determinants of health (SDOH)—the social and economic factors that influence an individual’s health outcomes [25]. Differences in income, education and locality (living in rural region, for example) affect a person’s access to economic and social resources that determine their ability to afford vaccination, understand health information, and access vaccination services. As a result, these determinants limit access for individuals to make healthy choices, such as HPV vaccination [25].

To increase HPV vaccination and immunity rates overall, priorities should be dedicated towards adolescent vaccination where the most benefits can be provided. Currently, HPV vaccination rates in the U.S. are increasing steadily, but are still far below the rates for other frequently recommended adolescent vaccines such as the Tdap (diphtheria, tetanus, acellular pertussis) and MenACWY (meningococcal disease) [26]. There are also vast disparities, regionally and by gender, for HPV vaccination rates. Some of the disparities between adolescent boys and girls can be explained by how vaccine recommendations evolved over time. Beginning in 2008, two years after the HPV vaccine was approved for use in the U.S., the CDC’s ACIP recommended vaccination for girls only [27]. In 2011, the ACIP updated its recommendations to include adolescent males, as well [28]. Misconceptions have persisted that the HPV vaccine is recommended for adolescent girls, only.

## REGIONAL DISPARITIES IN THE UNITED STATES

Within the U.S., HPV vaccination rates vary dramatically from state to state, contributing to unequal protection and disparities in adolescent and community wellbeing. In 2020, the CDC estimated that only 7 states had HPV vaccination series completion rates over 68%, compared to 12 states that had completion rates under 53% [29]. When viewing the distribution of completion rates by state, the states with the highest HPV series completion rates are concentrated in the Northeast, the wealthiest region in the U.S. [29]. Higher vaccination rates in these states lead to a reduced risk of preventable cancers and support better physical and psychosocial wellbeing outcomes over the life course. The states with the lowest HPV series completion rates, however, are concentrated in the South, the most impoverished region in the U.S. and the region where many health disparities, including higher rates of STIs and HIV occur [30]. Therefore, lower coverage in these settings may exacerbate existing wellbeing inequities by increasing exposure to preventable diseases. As of 2022, only 58.6% of adolescents in the U.S. aged 13-15 had received the full series of HPV vaccines [31].

## GLOBAL DISPARITIES AND STRUCTURAL BARRIERS

At the global level, HPV vaccination rates are tied to country wealth. LMICs have the highest incidence of HPV-attributable cancers [32], but also the world’s lowest HPV vaccination and screening rates. As of 2021, 85% of high-income World Health Organization (WHO) member countries had introduced national HPV vaccination programs, compared to only 30% of lower-middle-income countries [8]. These differences can be heavily attributed to the availability of educational, medical, and other supporting resources in LMICs. Specifically, the available routes of vaccine distribution play a large part in the differences in HPV vaccination rates between high- and low-income countries. In high-income countries, HPV vaccines programs are split between a mixture of school and facility-based sites [33]. In LMICs however, almost all HPV vaccination programs are school based. On one hand, school-based vaccination programs have been shown to achieve the highest completion rates for the HPV vaccine series [34]. For example, Rwanda’s HPV vaccination program achieved 93% for 3-dose coverage among girls in sixth grade through a school-based delivery model. The program had an active community engagement and a multi-phased national strategy that included catch up vaccination for older adolescent girls [35]. Similarly, other high-income countries like Australia, sent trained providers to schools through a government funded school-based vaccination program. This achieved higher vaccine uptake than alternative strategies such as school entry mandates or vaccine delivery through medical providers [36]. However, school-based programs are also more costly and require robust infrastructure for vaccines to be delivered to schools. For these reasons, school-based vaccination programs are more challenging for LMICs to sustain over a longer period of time. Improving vaccination coverage for adolescent HPV is complicated, however, without improved availability. Investing in public health infrastructure, including healthcare facilities, workforce capacity and coordinated delivery systems, is essential to allow an effective distribution of the HPV vaccine and improve overall wellbeing by preventing disease and reducing long-term health complications. In contrast, countries, facing infrastructural challenges, such as inadequate facilities, insufficient personnel and logistical barriers including limited storage and transportation capacity, often struggle to implement and sustain vaccination programs [37]. Both domestically in the U.S. and globally, HPV vaccination uptake worldwide will continue to lag behind public health goals.

## GLOBAL HPV VACCINATION POLICIES

The WHO’s 2020 Global Strategy to eliminate cervical cancer recommends including HPV vaccination in all national immunization programs and achieving 90% coverage among girls by age 15 by 2030 [8]. Achieving this goal requires securing affordable HPV vaccines through partnership and market-shaping strategies, expanding school- and community- based delivery, addressing vaccine hesitancy through effective communication, and updating national guidelines to reflect evidence that improves delivery efficiency [8]. The United Nations also set the Sustainable Development Goals (SDGs) as a global agenda to be achieved by 2030. Goal 3 focuses on ensuring healthy lives and promoting wellbeing for all ages. Its targets include achieving universal health coverage with financial risk protection, ensuring access to healthcare services, essential medicines and vaccines [38]. This goal also promotes research and development of vaccines and medicines for disease that disproportionally affect developing countries and supports their right to access affordable medicines under the Doha Declaration on the TRIPS Agreement and Public Health [38].

## CONCLUSION

Improving HPV vaccination coverage requires a multifaceted approach, combining public education, provider advocacy, and capacity building to ensure equitable access. Optimizing HPV vaccine uptake is a crucial strategy for enhancing long-term population wellbeing by reducing disease transmission, improving quality of life and building community resilience [39]. To remedy these disparities in uptake and increase vaccine uptake in general, countries must take several steps. To combat vaccine hesitancy and stigma, health providers can increase public education and recommendation of the vaccine; parents and healthcare providers can also support this goal through communication and advocacy [40]. Beyond education, capacity building is essential: expanding infrastructure, training providers, and ensuring sustainable supply chains. These efforts must be paired with increased research and targeted funding to develop distribution strategies tailored to local contexts whether addressing regional disparities in the U.S. or resource limitations in low- and middle-income countries. In addition, placing these strategies within a health equity framework may help explore beyond the individual behavior to address the social, economic, and structural factors that influence access to HPV vaccination, supporting more equitable and lasting improvements in vaccine uptake. By integrating education, accessibility, and equity-driven policies, we can significantly reduce HPV-related disease burden and move closer to global health goals.
